# Potential Diagnostic and Therapeutic Applications of Oligonucleotide Aptamers in Breast Cancer

**DOI:** 10.3390/ijms18091851

**Published:** 2017-08-25

**Authors:** Xiaoqiu Wu, Atik Badshah Shaikh, Yuanyuan Yu, Yongshu Li, Shuaijian Ni, Aiping Lu, Ge Zhang

**Affiliations:** Institute for Advancing Translational Medicine in Bone & Joint Diseases, School of Chinese Medicine, Hong Kong Baptist University (HKBU), Hong Kong 999077, China; isawu199205@gmail.com (X.W.); aatikshaikh@gmail.com (A.B.S.); yu.yy01@hotmail.com (Y.Y.); yongshuli000@163.com (Y.L.); jack6shuai@163.com (S.N.)

**Keywords:** aptamer, breast cancer, SELEX, diagnosis, targeted therapy

## Abstract

Breast cancer is one of the most common causes of cancer related deaths in women. Currently, with the development of early detection, increased social awareness and kinds of treatment options, survival rate has improved in nearly every type of breast cancer patients. However, about one third patients still have increased chances of recurrence within five years and the five-year relative survival rate in patients with metastasis is less than 30%. Breast cancer contains multiple subtypes. Each subtype could cause distinct clinical outcomes and systemic interventions. Thereby, new targeted therapies are of particular importance to solve this major clinical problem. Aptamers, often termed “chemical antibodies”, are functionally similar to antibodies and have demonstrated their superiority of recognizing target with high selectivity, affinity and stability. With these intrinsic properties, aptamers have been widely studied in cancer biology and some are in clinical trials. In this review, we will firstly discuss about the global impacts and mechanisms of breast cancer, then briefly highlight applications of aptamers that have been developed for breast cancer and finally summarize various challenges in clinical translation of aptamers.

## 1. Introduction

Breast cancer is a heterogeneous disease, which accounts for 29% of all new cancer cases and 14% of all cancer-related deaths among women [1]. Based on the detailed expression patterns and clinical implications, breast cancer could be divided into four subtypes: luminal A, luminal B, basal-like, and human epidermal growth factor receptor 2 (HER2) positive breast cancers [2,3,4]. Different subtypes of breast cancer exhibit distinct histopathological and biological features, which lead to differences in clinical outcomes and divergent responses to systemic interventions. Current treatment strategies for breast cancer consist of surgery, radiation therapy, chemotherapy and hormone therapy. Recent advances in early detection and breakthrough in treatment strategies has significantly improved survival rate in breast cancer patients. However, most breast cancer patients develop resistance to chemotherapy drugs, distant organ metastasis and recurrences, which reduce the prognosis of breast cancer. Thus, treating such patients poses a significant clinical challenge. In recent years, a better understanding of the molecular mechanisms underlying breast cancer has led to the identification of new molecular targets and development of targeted therapy. Targeting susceptible molecules in the pathways that prolong growth and promote invasion of cancer cells have spearheaded the discovery and clinical trials of several monoclonal antibodies (mAbs) and small-molecule inhibitors. Some of the targeted therapies widely used in breast cancer treatment include mAbs such as trastuzumab and pertuzumab; tyrosine kinase inhibitors such as lapatinib; cyclin-dependent kinase inhibitors such as palbociclib; mammalian target of rapamycin (mTOR) inhibitors such as everolimus; and poly-ADP-ribose polymerase (PARP) inhibitors [5]. Monoclonal antibodies are essential tools not only in biochemistry but also in molecular biology and medical research. They have revolutionized the traditional approach to treat serious diseases such as cancers and human immunodeficiency viruses (HIV). In addition, many mAbs are being actively evaluated in clinical trials [6]. Inevitably, there are various drawbacks that limit their clinical applications, such as difficulty in preparation, analysis, and transportation; easy degradation; and inflexible to chemical modification [7].

As an alternative molecular probe, aptamers offer significant advantages over existing mAbs [8,9,10,11], as illustrated in Table 1, such as small size, facile chemical synthesis easy modification [12], low-cost, low immunogenicity [13], rapid tissue penetration, high reproducibility and long-term stability [7,8]. Likewise, aptamers have better specificity and higher affinity towards the target molecule, have fewer non-specific cross-reactivities compared to antibodies [14,15,16,17,18,19], and have the capability to bind to a range of different molecules such as proteins, cells, tissues, or small molecules (organic dyes, amino acids, metal ions and nucleotides) [20]. In conjunction with the development of aptamer technology, aptamers have aroused wide public attention. In 2004, a vascular endothelial growth factor (VEGF)-specific aptamer, Macugen, was first approved by the Food and Drug Administration (FDA) for the treatment of neovascular (wet) age-related macular degeneration (AMD), which indeed has opened up new possibilities in the field of aptamers [21].

## 2. Breast Cancer

### 2.1. Current Status of Breast Cancer

Breast cancer is one of the most common cancers and the second leading cause of cancer associated deaths in women [22,23]. Currently, breast cancer accounts for 26% of all new cancer cases in women [22,24]. In 2016, approximately 246,660 women and 2600 men were expected to be diagnosed as new cases of invasive breast cancer in the United States [23,25]. Dramatic advances in early detection, better imaging techniques and breakthrough treatment strategies such as aggressive chemotherapy, hormonal therapy and targeted drug therapy have significantly improved the five-year survival rate among breast cancer patients [26,27]. However, breast cancer has high potential to undergo aggressive malignant transformation [28]. SEER staging reported that the overall five-year survival rates for localized and regional breast cancer patients are 99% and 85%, respectively, while, in breast cancer patients with distant metastasis, it is as low as 26% [29]. Additionally, approximately 30% of breast cancer patients reported recurrence within five years along with a 56% chance of metastasis, primarily to the bones, lung, brain, and liver [30].

### 2.2. Prognosis of Breast Cancer

#### 2.2.1. Subtypes of Breast Cancer

Breast cancer is a highly heterogeneous disease, which is composed of multiple subtypes based on molecular, histopathological and clinical levels. Each subtype has its own diverse prognostic and therapeutic implications [31]. Sørlie et al. pioneered studies into breast cancer classification and reported a distinctive “molecular portrait” of breast cancer using 456 cDNA clones. Accordingly, breast tumors were classified into five intrinsic subtypes with distinct clinical outcomes: luminal A (estrogen receptor (ER)+, progesterone receptor (PR)+, HER2− and Ki67 < 14%), luminal B (ER+, PR+, HER2− and Ki67 ≥ 14% or ER+, PR+, and HER2+), HER2-enriched (HER2+, ER− and PR−), basal-like (ER−, PR− and HER2−) and normal-like tumors [2,3,4,32]. This molecular classification has been shown to have significant prognostic value and acts as a predictive guide of the chemotherapy response [33]. Therefore, factors that influence survival and prognosis of breast cancer, including stages of cancer, tumor grade, age, histological subtype, hormone receptor status and HER2 receptor status, are helpful in the classification process [34].

#### 2.2.2. Breast Cancer Mechanism

Both genetic as well as lifestyle factors are implicated in breast cancer etiology. This includes lifestyle factors such as alcohol intake, combined treatment of estrogen with progestin, postmenopausal hormone therapy, leanness in early life followed by obesity in later life, lack of physical inactivity, use of oral contraceptives and exposure to ionizing radiation [35]. Approximately 70% of breast cancers with positive ERα expression belong to molecular subtype luminal A or luminal B, which strongly suggests that estrogen plays a pivotal role in survival, progression and development of breast cancer [36]. Mutation or deregulation in breast cancer susceptibility gene 1 (*BRCA1*) and breast cancer susceptibility gene 2 (*BRCA2*) has been implicated in familial breast cancer and women with first degree relative. Besides, the risk of breast cancer increases by two folds in women with first degree relative with breast cancer compared to women without any family history. Other risk factors for breast cancer are reproductive factors such as early age at menarche, late age at first birth, nulliparity and late age at menopause.

HER2 is found to be over expressed in several breast cancers and correlates with tumor size and estrogen expression. Additionally, HER2 expression was reported to be amplified in 20–25% of breast cancers and acted as an indicator of poor prognosis [37]. On the other hand, mutations, aberrant or deregulated expression of *TP53*, *MDM2* and *RB* genes have also been implicated to play a key role in therapeutic responses to breast cancer [38]. It is reported that restoration of functional activity of TP53 in TP53 deficient cells could sensitize these cells to chemotherapy drugs [39]. Several other genes are also involved in signaling and DNA repair defect in breast cancer such as fanconi anemia (FA) genes (*FANCD2*, *FANCA*, *FANCC*), mismatch repair endonuclease PMS2, mismatch repair genes (MutL homolog 1 (*MLH1*)), ataxia-telangiectasia, mutated (*ATM*) and Phosphatidylinositol-4,5-bisphosphate 3-kinase catalytic subunit alpha (*PIK3CA*) genes [40,41]. Further studies are being carried out to have a better understanding of breast cancer mechanism.

Triple negative breast cancer (TNBC) belongs to basal-like breast cancer that is immune-biologically negative for the ER/PR and HER2 protein expression. TNBC accounts for approximately 15% of the total newly diagnosed breast cancer patients and are usually found predominantly in younger patients. TNBC is more aggressive in nature in comparison to other subtypes and is characterized by high tumor grade, larger tumor size, early incidences of recurrence, higher tendency of early visceral metastases and lower median survival rate of only 10 months [42]. The present treatments available to TNBC patients are limited to surgery and aggressive chemotherapy [42]. On the other hand, the low percentage of survival rate among TNBC patients even after aggressive chemotherapy is attributed to chemo resistance. Over the past decade, several mechanisms involved in development of chemo-resistance in TNBC have been discovered: (1) efflux of chemotherapeutic drugs by cells which have overexpressed ABC transporter; (2) overexpression of β-tubulin III subunit; (3) mutations in DNA repair enzymes and DNA mismatch repair enzymes; (4) alterations in apoptotic genes (p53, caspase-3s, bcl-2, and bcl-x); (5) overexpression of ALDH1, raised glutathione and glutathione-S-transferase activity results in increased breakdown of chemotherapy agent such as cisplatin; and (6) nuclear factor (NF)-κB signaling pathways [43]. Currently, there are no targeted therapies available for TNBC treatment due to absence of targets such as hormonal receptors and HER2. Therefore, new tools are of high importance to be discovered for targeted therapy in TNBC patients.

## 3. Aptamer and SELEX

### 3.1. Aptamers

Early breast cancer detection and accurate diagnosis are vital for determining treatment strategy in breast cancer [44]. To accurately detect cancer-associated proteins, small molecules and cancer cells, present diagnostic tests mainly rely on antibody-antigen binding assays [45]. Unfortunately, these antibody based molecular tools often lack ideal pharmacokinetics and batch to batch reproduction, and easily lose their activity during chemical modification and storage [8].

In recent past, a new class of nucleic acid probes known as aptamers have gained increasing research interest as a molecular beacon. Aptamer, a term derived from the Latin aptus meaning “to fit” and the Greek word meros (“part”) [46], are short single-stranded DNA (ssDNA) or RNA oligonucleotides. By folding into distinct three-dimensional (3D) structures, aptamers can recognize the target with high specificity and sensitivity. As a molecular probe, aptamer has been integrated and utilized in bioanalysis and biomedicine [47].

**Figure 1:**
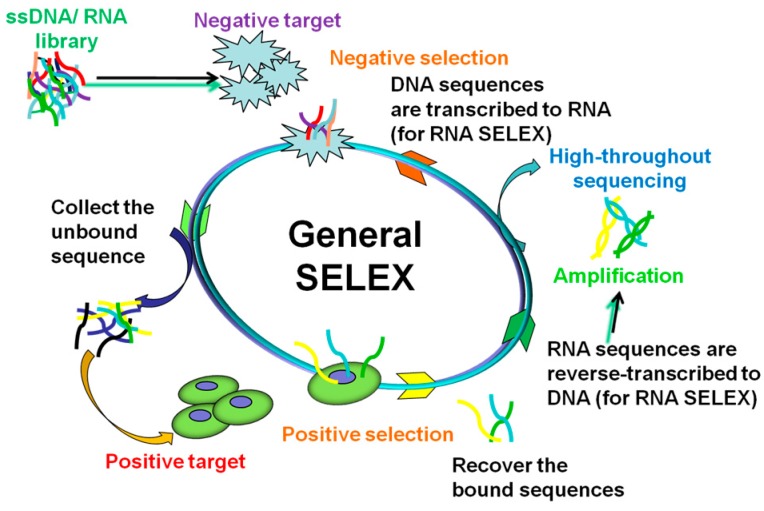
General SELEX procedures.

Aptamers are generated through an in vitro iterative selection process known as Systematic Evolution of Ligands by EXponential enrichment (SELEX) [46,48]. The traditional SELEX procedures are illustrated in Figure 1. Generally, DNA SELEX process includes three steps. In the first step, an ssDNA library will be synthesized, the library consists of primers at two terminals and a random sequence usually 30–40 mers in the middle [49]. The library contains as many as 1 × 10^13^ to 1 × 10^15^ different members. The second is the binding and separation step, in which target-bound sequences are separated from the unbound library. This step is essential for a successful aptamer screening. To improve the specific binding, counter-target will be added and only the target-bound library will be collected for the next step. The last step is amplification: the mixture from the second step will be used as a template for polymerase chain reaction (PCR) to create a new library for the next round of selection. The library of ssDNA is often prepared by the strand separation of PCR products. In the case of RNA SELEX, the complex recovered from the second step is reverse-transcribed to DNA, which will be used as template for PCR. Then, the PCR product is transcribed to RNA for another round. There is not much difference between DNA aptamers and RNA aptamers in functions. However, each has distinct advantages: DNA aptamers are more stable and much easier to be manufactured at a lower cost than RNA aptamers, while RNA aptamers have more diverse 3D conformations that can form complex structure to increase the binding specificity and sensitivity [49].

### 3.2. The Development of SELEX

Over the past two decades, SELEX has undergone rapid growth and led to development of a variety of screening methods such as Complex target SELEX [50], affinity chromatography SELEX [51], Tissue slide-based SELEX [52], Capillary electrophoresis SELEX [53], genomic SELEX [54], monoclonal surface display SELEX (MSD-SELEX) [55], and Cell-SELEX [56]. Along with these, several other new methods were derived from Cell-SELEX such as target expressed on cell surface-SELEX (TECS-SELEX) [57], fluorescence-activated cell sorting SELEX (FACS-SELEX) [16], 3D Cell-SELEX [58] and Hybrid-SELEX [59]. It is worth mentioning protein-based-SELEX and cell-based-SELEX, as these technologies are widely used in screening aptamers for breast cancer.

### 3.3. Protein-Based-SELEX

Protein-based-SELEX is a method for identifying new aptamers for a known purified intracellular or extracellular protein [60]. In general, target proteins are immobilized on the surface of beads and then incubated with the library. After washing, the protein-library mixture is used for PCR amplification (for ssDNA) or RT-PCR (for RNA) [61]. After about 20 rounds of selection, the evolutionary library is sequenced.

### 3.4. Cell-Based-SELEX

Cell-SELEX is aimed at developing a novel aptamer for the whole cell. The main steps of Cell-SELEX are similar to traditional SELEX, which includes incubation, partitioning and amplification. To generate aptamers that can specifically bind to target cells, a library is synthesized. The pool is incubated with the target cells as positive selection, then the unbound sequences are washed away and only the bound library is collected. Further, negative selection is carried out by incubating the recovered bound sequences with negative control cells, and only the unbound library is recovered for PCR [62]. In the case of RNA SELEX, the single-stranded RNA library is prepared by in vitro transcription of dsDNA templates, usually using recombinant T7 RNA polymerase. In DNA SELEX, the library is often prepared by PCR products. After 20 rounds of selection, the evolutionary library is sequenced by high-throughput sequencing [63].

Compared to protein-based-SELEX, cell-based-SELEX could discover novel targets without prior knowledge of the cell surface, which is quite essential to discover new biomarkers for treating diseases. In addition, some proteins only function in their native conformation, so these proteins cannot be used as targets for new aptamers by protein-based-SELEX [49].

## 4. Applications of Aptamer in Breast Cancer

Aptamers are an ideal molecular probe candidate due to its unique characteristics of high sensitivity and specificity towards a selected target. The use of aptamers as therapeutic agent was first demonstrated by Sullenger et al. in 1990 [64]. In the flowing part, we will explore diagnostic and therapeutic applications of aptamers against breast cancer. Aptamers that target breast cancer cells or proteins, since 2006 till today are listed in Table 2.

### 4.1. Aptamer-Based Diagnostic Applications

#### 4.1.1. Aptamers Bind to Targets Specifically

HER2, a receptor tyrosine-protein kinase, is highly over expressed in 20–30% of breast cancers [82]. HER2 positive breast cancer is one of toughest subtypes of breast cancer and associated with aggressive behaviors and poor clinical outcomes [83]. Likewise, HER2 is also a key prognostic marker and an effective therapeutic target. Therefore, it is essential to realize the early detection of HER2. At present, immunohistochemistry (IHC), chromogenic in situ hybridization (CISH) and fluorescent in situ hybridization (FISH) are three most commonly used methods in the clinical evaluation of HER2 expression in breast cancer tissue samples. IHC is often utilized as the screening test to detect the expression levels of HER2 protein, while CISH and FISH are used to detect *HER2* gene expression. In some ambiguous cases, the IHC results need further validation and confirmation by FISH, which is a more sensitive and reliable test [84]. However, these diagnostic technologies have serious weakness such as expensive equipment, difficulty in probe preparation and high technical requirements as an operator. Hence, they are not popular in general clinical laboratories. On the other hand, most of the breast cancer patients are generally diagnosed with the advanced or unresectable stage due to the lack of early detection tests and absence of recognizable signs or symptoms in localized disease state. Under this circumstance, there is a sense of urgency to develop novel, simple and rapid detection technology at the early stages [85].

Considering the importance of HER2 expression in breast cancer, Gijs et al. generated two novel DNA aptamers, HeA2_1 and HeA2_3, that target HER2 through an adherent whole-Cell SELEX strategy [65]. Both these aptamers could bind to HER2-overexpressing cells SKOV3 and SKBR3 with high specificity. Further, in vivo tumor tissue staining studies demonstrated a bright fluorescent staining for HeA2_1 and HeA2_3 aptamers on SKOV3 tumor tissue compared to no staining seen on HER2 negative MDA-MB-231 tumor tissue section. Aptamer HeA2_3 could also inhibit cancer cell proliferation, which will be further elaborately represented in the aptamers as drugs section.

Similarly, Kang et al. isolated a RNA aptamer SE15-8 that could specifically target extracellular domain of HER2 protein by cell-SELEX [77]. This RNA aptamer could bind with HER2 positive cell line MDA-MB-453 and KPL-4 but had no affinity towards HER2 negative cells such as MCF-7 and A431. In another study, Sett et al. reported the isolation of DNA aptamer ECD_Apt1 to specifically target extracellular domain of HER2 protein [67] and then conjugated the ECD_Apt1 aptamer with biotin. This biotin-aptamer conjugate showed stronger cytoplasmic staining in SKBR3 compared to MDA-MB-231 and MCF-7. Further, on breast cancer tissue, it showed specific and selective localization in the cytoplasmic niche of malignant ducts of cancer cells. Differently, Chu et al. compared specificity of DNA aptamers HB5 (target for HER2) to commercial anti-HER2 mAbs on 214 breast cancer samples by IHC in a clinical setting. Surprisingly, DNA aptamer HB5 displayed stronger membrane staining than the corresponding antibody [86]. Later studies showed that HB5 could also displayed relatively strong binding to SK-BR-3 and weak binding to MDA-MB-231.

In order to detect breast cancer tissue with metastasis Liu et al. [81] identified a high affinity DNA aptamer LXL-1-A that could bind to MDA-MB-231 cells which were derived from metastatic site and pleural effusion. The DNA aptamer LXL-1-A showed high specificity towards metastatic as well as tumor tissue and positively identified breast cancer tissue with metastasis in 76% of the cases.

The above findings suggest that aptamers could be generated to specifically target not just HER2 expressing cells but also positive primary and metastatic tumor tissue. Aptamers displayed better binding capability than corresponding antibody, thus could be used as an ideal candidate to design early stage detection system.

#### 4.1.2. Aptamers Bind to Targets with High Sensitivity

VEGF165 and MUC1 are known to play key roles in breast cancer. The aptamers of MUC1 and VEGF (AptMUC1 and AptVEGF) present high affinity to the corresponding proteins [87,88,89]. Wang et al. designed a new sensitive colorimetric assay by combining nanotechnology with these two aptamers [90]. Briefly, MUC1 binding aptamer linked to Pt-Au nanoparticle acted as a bio sensing probe towards MUC1. VEGF123 binding aptamer modified on magnetic beads acted as capture probe towards VEGF123. MCF-7 cells were used as target cell line for its high expression of MUC1 and VEGF165. This study demonstrated that Pt-Au nanoparticle-aptamer complex could detect MCF-7 cells with high sensitivity; it could detect as low as 10 cells/mL. This dual-aptamer targeted strategy could successfully distinguish between different cancer cells types and even from normal cells.

In another study, Kim et al. generated a novel RNA aptamer SE15-8 towards both extracellular domain of HER2 protein and HER2 positive breast cancer cells [66]. Zhu et al. developed an electrochemical immune sensor by using the above RNA aptamer SE15-8 and gold nanoparticles (AuNPs) [91]. They used glassy carbon electrode (GCE) as a fixed plate, the proposed sensor could positively distinguish HER2 and differentiated between HER2-positive breast cancer cells from HER2-negative cells. This method exhibited an excellent diagnosis method for the ultrasensitive detection of SK-BR-3 breast cancer cells in human serum samples with a minimum detection limit of 26 cells/mL. Thus, by using such method, a very simple, rapid, and sensitive diagnostic technology could be introduced for diagnosis of early stages of breast cancer and detection of disease progression and prognosis.

### 4.2. Aptamer-Based Therapeutics and Drug Discovery

The goal of anticancer therapy is to improve overall survival rate and reduce adverse effects. To achieve this goal, development of active tumor targeting system that delivers therapeutic agents selectively to cancer cells is necessary. Target oriented specificity and drug delivering capability are two vital aspects that need to be examined while developing active tumor targeting system. Although, the introduction of newer and better chemotherapy drugs has significantly improved the survival rate in some breast cancer patients. Inevitably, chemotherapy associated drawbacks such as drug adverse effects, poor tissue selectivity, low intra-tumoral accumulation, rapid systemic clearance and drug resistance have raised serious concerns [92].

**Figure 2:**
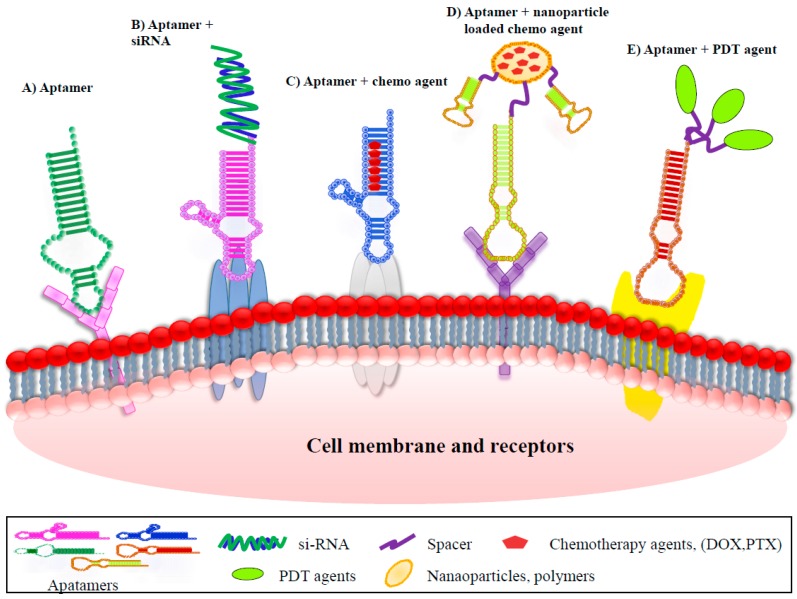
Therapeutic applications of aptamers for breast cancer.

Antibodies are the most widely used targeted ligands by virtue of their high specificity and ample availability. However, they have been found to elicit immunogenicity [93]. Therefore, aptamers with their ability to efficiently target specific cancer cells and tissue are being given due consideration as an alternative to existing antibodies. Aptamer-mediated active tumor targeted therapy could be classified into eight groups: aptamers as drugs, aptamer–small interfering RNA (siRNA) conjugates, aptamer–locked nucleic acid (LNA) conjugates, aptamer–anti-microRNAs (miRNAs) conjugates, aptamer–chemotherapy agent conjugates, aptamer–nanoparticles (NPs) conjugates, aptamer–photo dynamic therapy (PDT) agent conjugates, aptamer-mediated immunotherapy. General paradigms that have appeared most frequently in the recent literatures are graphically represented in Figure 2.

#### 4.2.1. Aptamers as Drugs

Currently, trastuzumab and pertuzumab are widely used in the treatment of HER2 positive breast cancers [94,95]. Unfortunately, one of the most important shortcomings for this treatment is the development of resistance to this therapy [96]. This has highlighted aptamer as an attractive alternative.

To study the anti-tumor activity of a DNA aptamer, Gijs et al. identified a relatively short (40-mer) DNA aptamer HeA2_3 as reported earlier and investigated its efficacy in inhibiting cancer cell growth in vivo. They reported that the aptamer HeA2_3 could actively identify HER2 overexpressing cancer cells, undergo internalization via receptor mediated endocytosis and then inhibit cell progression, growth and viability [65]. This finding may be useful to design aptamer-mediated delivery of drugs, siRNA and radionuclides.

Periostin is another important extracellular matrix protein associated with cell proliferation and metastasis. It is found to be over expressed in several human cancers especially breast cancer. In order to specifically target periostin and inhibit its functions, Lee et al. generated a DNA aptamer, PNDA-3 [76]. PNDA-3 aptamer could efficiently bind to periostin-expressing cells 4T1 but not to periostin-deficient cells MCF-7. In vitro studies showed that PNDA-3 aptamer could markedly antagonize the periostin-induced adhesion, migration, and invasion of breast cancer cells and block the activation of various components of the αvβ3 and αvβ5 integrin signal transduction pathways. Moreover, PNDA-3 aptamer significantly reduce primary tumor growth and prevent distant metastasis in tumor mouse model.

Hence, aptamers such as HeA2_3 and PNDA-3 could be integrated as a potential targeted therapy against breast cancer (Figure 2A).

Notably, in TNBC the epidermal growth factor receptor (EGFR) is frequently overexpressed. EGFR is a 170 kD integral membrane protein, characterized by tyrosine kinase activity through which it exerts control of key cellular transduction pathways in both normal and cancerous cells. Increased expression of EGFR was seen in a variety of tumors such as breast, lung, colorectal, prostate and bladder cancer. Besides, EGFR overexpression is associated with poor prognosis [97]. Presently, anti-EGFR targeted therapies including mAbs, e.g., cetuximab and panitumumab, and small molecule tyrosine kinase inhibitors (TKIs), e.g., afatinib, are approved for treatment of non-small cell lung cancer (NSCLC) and colorectal cancer (CRC). However, due to disappointing results of TKIs and mAbs as monotherapy and in combination against breast cancer, they have not been approved for treatment of TNBC [98]. To overcome this challenge Camorani et al. developed a nuclease resistant RNA-aptamer CL4 that had high affinity towards the extracellular domain IV of human EGFR. They reported significantly inhibition of tube formation in TNBC-like cells. In addition, CL4 aptamer when injected in a mouse xenograft model resulted in a strong inhibition of tumor growth [99].

The first aptamer drug, Macugen, was approved by US FDA in December 2004 to treat age-related macular degeneration [21,100]. Macugen is a PEGylated RNA aptamer that targets VEGF 165 which plays a vital role in angiogenesis and permeability [101]. In addition, aptamer based targeted therapy against various diseases are under development and a few have entered the clinical trial stage (shown in Table 3) [102,103,104,105,106,107,108,109,110].

#### 4.2.2. Aptamer–Small Interfering RNA(siRNA) Conjugates

*Bcl-2* is an anti-apoptotic gene. The elevated expression of Bcl2 protein could improve the survival of HER2 positive cancer cells [111,112,113]. Aptamers have an important characteristic that they could easily undergo chemical modification (Figure 2B). Thiel et al. exploited this property to conjugate aptamers with si-RNA. Firstly, they isolated a novel RNA aptamer by cell-internalization SELEX protocol towards HER2 positive cells. The developed aptamer could be internalized as well as could deliver therapeutic si-RNA into the cytoplasm [80]. They covalently linked this aptamers to si-RNA which targeted Bcl-2 and then showed that when HER2 aptamers–Bcl-2-si-RNA conjugate was applied to different types of cell lines; the conjugates could successfully internalize into HER2 positive cells and thereby silence *Bcl-2* gene expression. Thus, silencing of *Bcl-2* could expose the HER2 positive cells to become more sensitive to chemotherapy drugs.

#### 4.2.3. Aptamer–Locked Nucleic Acid (LNA) Conjugates

LNA is a modified RNA nucleotide with a special conformation. This structure can increase binding affinity and remarkable nuclease resistance. Nonaka et al. developed a novel DNA aptamer Vap7 aimed against the receptor-binding domain (RBD) of VEGF with high affinity [75]. Edwards et al. further modified Vap7 aptamer with a LNA to form a complex named RNV66. This RNV66 complex could effectively suppress breast cancer cell proliferation in vitro and in vivo experiments. Based on the results, we believe that RNV66 could be used as a potential therapeutic candidate that offers great promise for future pre-clinical investigations [114].

#### 4.2.4. Aptamer–Anti-MicroRNAs (miRNAs) Conjugates

miRNAs are small non-coding RNA molecules (containing 19–25 nucleotides) that function in RNA silencing and post-transcriptional regulation of gene expression [115]. Shu et al. developed a multifunctional RNA conjugate complex (3WJ–aptamer–anti-miRNA conjugate), in which CL4 aptamer functioned as a EGFR targeting agent, anti-miR-21 RNA acted as a therapeutic agent, and a three-way junction (3WJ) motif supported as a molecular scaffold. They demonstrated that systemic administration of 3WJ-EGFRapt/anti-miR-21 nanoparticles injection in TNBC tumor bearing mice could efficiently be internalized into tumor cells then inhibit miR-21 and thus result in suppression of tumor growth [116].

#### 4.2.5. Aptamer–Chemotherapy Agent Conjugates

Doxorubicin (Dox) is one of the most potent chemotherapeutic drugs, which has shown great treatment potential against variety of cancers. However, its wide range of clinical applications was hampered due to emergence of drug resistance and serious cardiotoxicity [117,118]. By understanding the modification potential of aptamers, several researchers devised a strategy to conjugate chemotherapeutic drugs to tumor-targeting aptamers. This drug–aptamer conjugate increase dosage of drug delivery to specific target cancer cells and minimize the exposure to non-target sites (Figure 2C). Liu et al. generated a new DNA aptamer (HB5) using an adherent whole-cell SELEX approach. HB5 could selectively bind to both ECD of HER2 and HER2 enriched breast cancer cells [68]. They formulated Apt-Dox complex by intercalating Dox into the DNA structure and their further studies demonstrated that Apt-Dox complex could selectively deliver doxorubicin to HER2 overexpressed breast cancer cells sparing HER2 negative cells. Importantly, HB5 can increase the cytotoxicity and reduce the tumor cell growth.

MUC1, is a large transmembrane mucin glycoprotein. Its expression was reported to be increased at least 10-fold in several cancers such as adenocarcinoma, lung cancers, colon cancers and breast cancers, thus making MUC1 protein an attractive anti tumors target [74]. Hu et al. initially selected a DNA aptamer MA3 that targeted a peptide epitope of MUC1 and then conjugated the MA3 aptamer with Dox to form an Apt-Dox complex. This Apt-Dox complex had shown preferentially binding to MUC1 enriched cells MCF7 and effectively deliver DOX into the target cells but not MUC1-negative cells [74]. Hence, this strategy of targeting ligands to selectively deliver cytotoxic drugs (Figure 2D) shows the plausible role of aptamer–drug conjugate in targeted therapy.

#### 4.2.6. Aptamer–Nanoparticles (NPs) Conjugates

NPs are particles 1–100 nanometers (nm) in size. NPs are of great scientific interest, as they have proven to have great potential in drug delivery due to their passive tumor-targeting effect of enhanced permeability and higher retention (EPR) on solid tumors. [119]. Paclitaxel (PTX), a natural taxane diterpene, is widely used as a chemotherapy drug as monotherapy or in combination with other drugs [120]. Aptamer-mediated nano therapeutics assist in bypassing pathophysiological barriers to boost uptake and greater retention of cytotoxic drugs by target cells. Yu et al. developed a novel drug delivery system. They firstly formulated a PTX loaded poly (lactic-co-glycolic-acid) (PLGA) nanoparticles complex and conjugated these nanoparticle to MUC1 protein targeted DNA aptamer S2.2 via a DNA spacer [121]. They reported that MCF-7 cancer cells had an increased uptake of PTX loaded Apt-NPs, with enhanced cytotoxicity.

Mahlknecht et al. selected a new DNA aptamer 2-2 for HER2 using extracts of N87 cells. Serious experiments with DNA aptamer 2-2 for HER2 illustrated that the trimeric version of this aptamer could promote the translocation of HER2 from the cell membrane to the cytoplasm, then enhance the degradation of HER2 and inhibit tumor proliferation [122]. A serious disadvantage of nanoparticle is that they cannot escape from vesicle after endocytosis and thus induce a biological response. Lee et al. [123] exploited this disadvantage by conjugating gold nano-particles with anti-HER2 DNA aptamer 2.2 (HApt-AuNs complex). They demonstrated that this complex could specifically target HER2 positive cells and are internalized. Apt-AuNS accumulates in the lysosomes, thereby promoting lysosomal degradation of HER2 and ultimately triggering cell death and cell cycle arrest.

#### 4.2.7. Aptamer–Photo Dynamic Therapy (PDT) Agent Conjugates

PDT is a minimally invasive technique and has been approved for treatment of several cancers such as esophagus, skin, tirades, and respiratory system [124]. PDT has several advantages including low toxicity, repeatedly and immunological effects. PDT as treatment of breast cancer is still in initial stages [124]. Wu et al. conjugated a DNA aptamer S2.2 with Ag–Au nanoparticles. These Apt–Ag–Au nanostructures demonstrated high affinity and specificity to recognize MUC1 protein on the surface of MCF-7. In addition, these Apt–Ag–Au nanostructures have high capability to adsorb near-infrared (NIR) irradiation. Hence, photo thermal therapy of MCF-7 cells could be performed at a very low irradiation power density without any harm to healthy cells [125] (Figure 2E).

On similar note, Ferreira et al. identified a special DNA aptamer 5TR1 targeting MUC1 [73]. It can bind specifically to cells expressing MUC1 glycoforms as well as MUC1+ epithelial cancer cell lines such as MCF-7. Chlorin e6 plays a key role in the photodynamic therapy. In this study, they modified chlorin e6 at 5′ end of aptamer 5TR1 and demonstrated that a remarkable enhancement (>500-fold increase) in toxicity upon light activation was achieved compared to the drug alone.

Shieh et al. used dimeric G-quadruplexed AS1411 aptamer that has high affinity and specificity towards nucleolin. The specialized dimeric G-quadruplex structure of the AS1411 aptamer could non-covalently bound with several molecules of TMPyP4 (a porphyrin). The results showed that Apt-TMPyP4 complex derivative resulted in higher accumulation of TMPyP4 in nucleolin positive MCF-7 cells, and had significantly higher photo-damage in MCF-7 cells compared to normal epithelium cells when explosive to light [126] (Figure 2E). Development of these types of receptors-targeted aptamers conjugates with photosensitive molecules could highlight their potential in discovery of more efficient cancer photo chemotherapy agents.

#### 4.2.8. Aptamer–Mediated Immunotherapy

Immunotherapy, a strategy that harnesses immune system to combat tumors was chosen as the breakthrough of 2013 [127]. T cells are the final effectors of immune-mediated cancer regression; strategies that directly use tumor-reactive T cells as an immunotherapy have been developed [128]. Memory T cells plays an important role in developing protective immunity against pathogens and cancers. Differentiation of activated CD8+ T cells into protective memory cells can be promoted by inhibiting key regulators. This led to the discovery of several immune-stimulatory antibodies against co-inhibitory receptor cytotoxic T lymphocyte antigen 4 (CTLA4), such as ipilumimab. CTLA-4 is an inhibitory receptor that is over expressed only on advanced CD4 and CD8 cells [129]. However, apart from showing positive clinical benefits, these antibodies face serious dose limiting toxicities and adverse effects in clinical trials. This hurdle paved way for the development of aptamers [130]. The first aptamer was developed to bind to CTLA-4 [131]. Gilboa et al. demonstrated that this nuclease resistant aptamer could reserve CTLA-4 mediated inhibition of T cells and the tetravalent form of this aptamer also can induce protective immunity in mouse tumor models. 4-1BB is a major costimulatory receptor promoting the survival and expansion of activated T cells [132]. McNamara et al. generated an aptamer targeted towards 4-1BB based on the effectives of agonistic anti-4-1BB antibody. They showed that the 4-1BB aptamer could not only cause T cells activation but also induce tumor rejection in mice tumors [133]. Further, Benaduce and coworkers’ study on 4-1BB aptamer, showed that 4-1BB aptamer could achieve similar tumors suppression as that of 4-1BB mAbs and has superior therapeutic index over mAbs [134]. Consequently, aptamer application in immunotherapy is limited as it targets only costimulatory ligands that are mostly expressed on surface of tumor or immune cells and does not undergo internalization upon ligand binding.

### 4.3. Molecular-Imaging

Current clinical imaging modalities used in breast cancer detection include nuclear imaging such as positron emission tomography (PET), single photon emission computed tomography (SPECT), conventional radiological modalities such as magnetic resonance imaging (MRI), computed tomography (CT) and ultrasonography (USG) and optical imaging. However, each imaging modality has its own pros and cons. At present, the achievement of multimodal molecular imaging mainly relies on the development of multi-functional molecular imaging probes.

In 2010, Li et al. developed a nucleolin-targeted optical/magnetic resonance (MR) dual imaging nanoprobe by covalent coupling reaction between PEG-Gd2O3 NPs and AS1411-Ag NCs to track cancer cells [135]. Due to high expression of nucleolin, MCF-7 cells were used as the target. This nanoprobe could easily recognize MCF-7 cells, which was demonstrated by bright fluorescence signal, while no fluorescence signal was observed in the case of NIH-3T3 mouse fibroblast cells. Therefore, nucleolin positive tumor cells could be specifically tracked by both fluorescence imaging and magnetic resonance imaging in vitro. Thus, aptamer could be incorporated for tumor imaging in the future.

## 5. Challenges for Aptamers in In Vivo Therapeutic Trials and Solutions

Aptamers, functionally comparable to traditional antibodies, offer several advantages, as mentioned earlier. However, even after a few aptamers underwent clinical trials, only one aptamer drug was approved by FDA. As nucleic acid biopolymers, the intrinsic physicochemical feature poses serious challenges for aptamers as shown in Table 4. These limitations have delayed or even suppressed the clinical translation of aptamers.

### 5.1. Nuclease-Mediated Degradation

Aptamers composed of unmodified nucleotides have half-lives as short as 10 min in the blood due to the activity of endogenous nucleases [136]. Chemical modifications need to be used to increase serum half-life; most aptamers in clinical studies have been chemically modified. Modifications can be introduced during the SELEX or after discovery. Modifications with 2′-aminopyrimidines, 2′-fluoropyrimidines, 2′-Omethylnucleotides and LNA have been successfully incorporated into the DNA/RNA library [137,138,139]. Multiple modifications could be used after SELEX. Most of the post-SELEX modifications could be advantageous, while some might affect the inherent properties and change the structures of the original aptamers. Therefore, it is necessary to precisely tailor the modifications for the desired functions [140].

### 5.2. Renal Filtration

The average diameter of aptamers with a mass of 6–30 kDa and molecular mass range (20–100 nucleotides long) is less than 5 nm [141]. Although the low molecular weight allows economical chemical synthesis and better target accessibility, it facilitates rapid excretion of non-formulated aptamers through renal filtration. Hence, as the molecular mass cut-off for the renal glomerulus is 30–50 kDa [49,110], aptamers are generally formulated with a bulky moiety by increasing the molecular weight to overcome renal filtration and extend its circulation time. The most commonly used polymer for preventing exclusion is high-molecular-mass polyethyleneglycol (PEG) [136]. PEGylation has been extensively used with proteins, peptides, small molecules, and oligonucleotides as a strategy for slowing down renal clearance and extending half-life [142]. Cholesterol conjugation has also been reported as an alternative strategy to reduce renal filtration rates, although the extent of this effect seems to be less than for PEG conjugation [110,139,143].

### 5.3. Toxicity

Because there is only one approved aptamer drug (Macugen), availability of toxicological information regarding aptamers in humans is very limited [144,145]. The toxicological testing of Macugen and anticancer aptamer AS1411 revealed very minimal toxicity [142,146]. Although aptamer-related adverse events are rare in clinical evaluations to date, potential toxicities may arise from polyanionic effects, unexpected tissue accumulation, intensive chemical modification or conjugation, nonspecific immune activation and in particular with continuous or repeated administration of aptamer therapeutics [147]. As with other therapeutics, aptamer toxicity can manifest itself through on-target or off-target mechanisms. In conjunction, highly negatively charged molecules are prone to nonspecific binding towards blood proteins, which may result in high uptake by non-target tissues and organs, thereby causing unwanted side effects and reducing therapeutic efficacy [148,149]. Chemical modifications have proved to be a double-edged sword, as unnatural nucleotides may cause chemical toxic effects or become immunogenic. Thus, chemical modifications should be used cautiously according to the desired therapeutic application of the aptamers. Adverse responses could also be associated with the formulation of therapeutic aptamers.

## 6. Future Perspectives

Aptamers can bind to their targets with high affinity and selectivity. This feature makes aptamers ideal candidates to be used as molecular probes in clinical research, especially in cancer studies. Besides the applications mentioned above, aptamers can also be used for biomarker discovery due to cell-SELEX is a method for generating specific aptamers targeting cancer cells without prior knowledge of the membrane proteins.

Biomarker discovery plays a vital role in the development of our knowledge about diseases. Molecular biomarkers cannot only detect pathogens but also can differentiate pathogenesis, they are unique measurable indicators for cellular state as well as disease screening, disease progression and treatment effectiveness [150]. Furthermore, newer biomarkers are useful for discovery and development of novel targeted therapy. There are relatively few biomarkers in clinical practice, and the main reason is the absence of efficient methods for biomarker discovery [151]. Among the latest technologies, aptamers generated by recent SELEX approach can specifically recognize cell-surface biomarkers without prior knowledge of their molecular signature.

At present, there are two groups are working on this field. The first group, Shangguan et al. had developed individual aptamers Sgc8, which can specifically identify CCRF-CEM cell line and then identified the target of sgc8 [152]. The main procedures they used include protein identification and Mass Spectrometry sequencing. Another group discovered new biomarkers by utilizing pools of aptamers instead of an accurate aptamer. This is termed as aptamer-facilitated biomarker discovery (AptaBiD) [153]. There are three main steps in this approach: the first step is to select an aptamer pool that can specifically bind to the target, then isolate the protein–aptamer pool mixture, and finally validate the accurate protein by mass spectrometry. In this study, live mature and immature dendritic cells are used as targets and six biomarkers that were previously unknown are identified.

Biomarkers are quite important for detailed classification and accurate targeted therapy. Thus, these new approaches could be similarly utilized in the discovery of new biomarkers for breast cancer. Although relevant applications are new, and still in developmental stage, with significant progress achieved, aptamers could play a vital role in breast cancer management in the future.

## 7. Conclusions

Breast cancer is heterogeneous in nature, at both epidemiological and molecular levels [154]. Despite the development in early detection and management of breast cancer patients, the prognosis of this disease remains a major public health problem and poses a significant challenge to clinicians [155]. With the existing therapeutic methods, five-year survival rate for early-stage breast cancer patients has increased to 99%, while for breast cancer patients with metastasis this rate is still below 30% [27]. Moreover, these therapeutic methods have serious side effects and have also resulted in drug resistance. Thus, there is desperate need to develop tailored and targeted therapy.

SELEX strategy provides an effective approach to screen aptamers that specifically aim at a variety of targets. As recognition probes, aptamers have several advantages including efficient and cost-effective chemical synthesis, easy and controllable modification with functional moieties to meet various clinical requirements, large-scale commercial production, non-toxicity, and limited immunogenicity [151,152,153]. Aptamers have been widely used in cancer cell biology, drug discovery [21], targeted drug delivery [63,154,155], molecular imaging [135], and several other fields.

Various applications of aptamer have been reported for breast cancer. For example, aptamer could promote the early detection of breast cancer due to its high sensitivity and specificity, thus it could be used as a new diagnostic tool. In addition, as a new targeted therapeutic agent, some aptamers could act as drugs by interacting with their target, other aptamers could be conjugated with si-RNA [80], LNA, miRNAs, chemotherapeutic drugs, PDT agents [125] or nanoparticles to formulate a new complex to specifically kill cancer cells without causing any harm to healthy cells. Besides, aptamers can also be conjugated with imaging agents to efficiently detect specific types of cancer and monitor drug efficacy and disease progression.

Application of aptamer technology in cancer diagnosis and therapy is still in its primal stage, and several challenges need to be overcome. For example, aptamer half-life in biological systems is relatively low, and unmodified aptamers are highly susceptible to degradation by endogenous nucleases [136]. Therefore, chemical modification is necessary to protect aptamers against nuclease degradation, reduce toxicity and also improve their half-life [141]. Furthermore, Macugen is the only aptamer that has been used as a drug to treat disease and most of the aptamers are still in clinical trials.

Aptamers have attractive biological functions and pharmacokinetic attributes for clinical applications in both cancer diagnosis and targeted cancer therapy. Hence, further investigation and improvements need to be conducted to facilitate clinical translation of aptamers mediated targeted cancer therapy.

## Figures and Tables

**Table 1:** Comparison between nucleic acid aptamers and monoclonal antibodies.

Features	Aptamers	mAbs
Basic composition	A, G, T/U, C/	Amino acid
Size and molecular weight	~2 nm; 6–30 kDa	~15 nm; 150–180 kDa
Synthesis time	Weeks	Months
Manufacture and cost	In vitro; cheap	In animals; expensive
Storage	Stable at ambient temperature	Low temperature
Target	Almost anything	Immunogenic targets
Immunogenicity	None or low immunogenicity	Significant immunogenicity
Specificity and affinity	High	High
Modification	Easy chemical modification	Hard to be modified
Reproducibility	None or low batch variation	Significant batch variation

**Table 2:** Aptamers that have been identified to breast cancer.

Target	Aptamer	Name	Reference
HER2 protein	DNA	HeA2_1, HeA2_3	[65]
HER2 protein	RNA	SE15-8	[66]
Extra-cellular domain (ECD) of HER2protein	DNA	ECD_Apt1	[67]
Epitope peptide of HER2	DNA	HB5	[68]
HER2 extracellular domain	DNA	Heraptamer1, Heraptamer2	[69]
HER2 protein	RNA	TSA14	[70]
HER2 peptide	DNA	HY6	[71]
ERα	DNA	ERaptD4	[72]
Cell surface associated (MUC1) protein	DNA	S2.2	[9]
Five MUC1 tandem repeats (MUC1-5TR)	DNA	5TR1	[73]
A peptide epitope of MUC1	DNA	MA3	[74]
Receptor-binding domain (RBD) of VEGF	DNA	Vap7	[75]
Periostin	DNA	PNDA-3	[76]
SK-BR-3 cell line	RNA	S6	[77]
MCF-10AT1 cell line	DNA	KMF2-1a	[78]
MCF-7 cell line	DNA	MS03	[79]
N202.1A cell line	RNA	C1,E1	[80]
MDA-MB-231 cell line	DNA	LXL-1	[81]
Breast cancer tissue	DNA	BC15	[52]

**Table 3:** Current status of 11 aptamers undergoing clinical trials.

Aptamer	Target	Conditions	Current Status	Reference
Pegaptanib (Macugen)	VEGF	Age related macular degeneration/diabetic macular edema/proliferative diabetic	FDA approved	[21]
NU172	Thrombin	Heart disease	Phase II	NCT00808964 *
AS1411 (AGRO-100)	Nucleolin	Renal cell carcinoma/non-small cell lung cancer	Awaiting Phase III	[103]
ARC1779	A1domain of von Willebrand factor	von Willebrand‘s disease	Awaiting Phase III	NCT00742612 *
ARC1905	Complement component 5 (C5)	Neovascular age related macular degeneration	Phase II/III, (recruiting)	NCT02686658 *
ARC19499 (BAX499)	Tissue factor pathway inhibitor (TFPI)	Hemophilia	Phase I/II	NCT01191372 *
REG1 (RB006 plus RB007)	Coagulation factor IXa	Coronary artery disease	Phase III	[102]
NOX-A12	Angiogenic chemokine (C-X-C motif) ligand 12 (CXCL1)	Tumor	Phase II	NCT01486797 *
NOX-E36	Pro-inflammatory chemokine C-C motif-ligand 2(CCL2)	Type II diabetes mellitus/Renal impairment/nephropathy/lupus nephritis	Phase II	NCT01547897 *
NOX-H94	Hepcidin	Anemia of chronic disease	Phase II	NCT01691040 *
E10030	Platelet-derived growth factor (PDGF)	Neovascular age related macular degeneration	Awaiting Phase III	NCT01944839 *

* clinical trial number.

**Table 4:** Challenges of aptamer therapeutics.

Challenge	Solution
Nucleases mediated degradation	Chemical modification
Rapid renal clearance (short half-lives)	Increasing the weight of aptamer by conjugating cholesterol or PEG
Cost of production	Decrease the usage of aptamer
Toxicity (nonspecific immune activation and polyanionic effects)	Cautious modification

## Abbreviations

ssDNA	Single-stranded DNA
3D	Three-dimensional
HER2	Human epidermal growth factor receptor 2
SELEX	Systematic evolution of ligands by exponential enrichment
FDA	Food and Drug Administration
mAbs	Monoclonal antibodies
NSCLC	Non-small cell lung cancer
CRC	Colorectal cancer
VEGF	Vascular endothelial growth factor
AMD	Age-related macular degeneration
ER	Estrogen receptor
PR	Progesterone receptor
ERα	Estrogen Receptor α
IHC	Immunehistochemistry
CISH	Chromogenic in situ hybridization
FISH	Fluorescent in situ hybridization
Dox	Doxorubicin
PLGA	Paclitaxel (PTX) loaded poly (lactic-co-glycolic-acid)
TNBC	Triple negative breast cancer
PDT	Photo dynamic therapy
PET	Positron emission tomography
SPECT	Single photon emission computed tomography
MRI	Magnetic resonance imaging
NIR	Near-infrared irradition
CT	Computed tomography
USG	Ultrasonography
ATM	Ataxia Telangiectasia Mutated
*BRCA-1*	Breast Cancer susceptibility gene 1
*BRCA-2*	Breast Cancer susceptibility gene 2
RBD	Receptor-binding domain
LNA	Locked nucleic acid
GCE	Glassy carbon electrode
ECD	Extra-cellular domain
FACS-SELEX	Fluorescence-activated cell sorting SELEX
MSD-SELEX	Monoclonal surface display SELEX
TECS-SELEX	Target expressed on cell surface-SELEX
MLH1	MutL homolog 1
Bcl-2	B-cell lymphoma 2
siRNA	Small interfering RNA
PARP	Poly-ADP-ribose polymerase
TKIs	Tyrosine kinase inhibitors
HIV	Human Immunodeficiency Viruses
AptaBiD	Aptamer-facilitated biomarker discovery
PIK3CA	Phosphatidylinositol-4,5-bisphosphate 3-kinase catalytic subunit alpha
TFPI	Tissue factor pathway inhibitor
NPs	Nanoparticles
PEG	Polyethyleneglycol
PTX	Paclitaxel
MUC1	Cell surface associated
PCR	Polymerase chain reaction
TNBC	Triple-negative breast cancer
3WJ	Three-way junction
CTLA4	Cytotoxic T lymphocyte antigen 4 CTLA4
nm	Nanometers
EGFR	Epidermal growth factor receptor
